# Total Play Time Needed for Preschoolers to Reach Recommended Amount of Non-Sedentary Activity

**DOI:** 10.3390/ijerph19063354

**Published:** 2022-03-12

**Authors:** Andrew E. Koepp, Elizabeth T. Gershoff, Darla M. Castelli, Amy E. Bryan

**Affiliations:** 1 Department of Human Development and Family Sciences, The University of Texas at Austin, Austin, TX 78712, USA; liz.gershoff@austin.utexas.edu (E.T.G.); aebryan@utexas.edu (A.E.B.); 2 Department of Kinesiology and Health Education, The University of Texas at Austin, Austin, TX 78712, USA; dcastelli@utexas.edu

**Keywords:** physical activity guidelines, early childhood, preschool

## Abstract

Health guidelines suggest that caregivers provide preschoolers with opportunities to be physically active for 3 h per day (roughly 15 min per waking hour), but because children are not continuously active, it is unclear what amount of time is needed to reach this goal. This naturalistic study enrolled 67 children (*M* = 4.5 years, 46% female) who wore accelerometers to measure their activity during indoor and outdoor free -play (*N* = 315,061 s). An hour of indoor play was insufficient for most children to reach 15 min of physical activity. When outside, most children reached 15 min of physical activity after slightly more than 30 min. Children engaged in outdoor activity sporadically (1.7 starts/stops per minute). Most physical activity occurred in bouts shorter than 20 s. Indoor free-play does not, on its own, provide sufficient opportunity for preschoolers to engage in physical activity consistent with health guidelines. As a result, outdoor play for at least 30 min at a time has a key role in meeting these guidelines.

## 1. Introduction

Regular physical activity promotes healthy development in preschool-aged children by preventing obesity [1], enhancing cardiovascular function [2], and building motor coordination [3]. Children under six years of age who are physically active show better weight status and bone health [4] as well as improved cognitive development and psychosocial health [5]. Given these health benefits, guidelines from the U.S. Department of Health and Human Services (USDHHS) [6] suggest that children should be physically active throughout the day and that adult caregivers should encourage active play across a range of activities. Guidelines from the Canadian Society of Exercise Physiology suggest that preschool-aged children engage in three hours of light, moderate, or vigorous physical activity per day [7]. In contrast to exercise, physical activity need not be structured and refers to any body movement that engages muscles and expends more energy than resting, reflecting a range of intensity that includes light, moderate, and vigorous activity [6]. The majority of preschool-aged children are enrolled in center-based care and this share is expected to increase over the next decade [8]. Because children may spend several hours per day in preschool or childcare, the Institute of Medicine (IOM) [1] recommends that educators provide opportunities for children to engage in physical activity for at least a quarter of the time that they are in care (i.e., 15 min per hour).

One way to promote physical activity is to allow children more time to play outdoors [9]. Because young children engage in physical activity through play, they do so with frequent starts and stops rather than through continuous activity [10]. This means that for children to reach the IOM recommendation of 15 min of activity, they likely need to engage in play for a period of time longer than 15 min. Despite these recommendations, many care settings do not provide adequate opportunities for physical activity [11] and young children remain overly sedentary and under-active [12]. A study of children in 125 Head Start centers across the United States found that 50% in full-day programs and 62% in half-day programs were allowed 30 min or less of outdoor play time; indeed, 10% of children in full-day programs and 13% of children in half-day programs were only allowed 15 min or less of outdoor time per day [13].

### 1.1. Physical Activity in Early Childhood Education Settings

Although young children are naturally physically active, the amount of physical activity that they engage in depends on the environment and the opportunities adults caregivers create for them [14,15,16]. Because children’s behavior is influenced by their immediate contexts [17], their play and activity differs according to indoor and outdoor settings, which are set up to promote different kinds of play. In early childhood education, indoor play is typically organized around different centers, with sedentary activities that foster creative exploration (e.g., drawing), fine motor development (e.g., puzzles), and pre-literacy skills (e.g., reading center). Although some activity centers may involve movement (e.g., block building or dramatic play), it is typically within a restricted range.

In contrast, outdoor spaces in early childhood education are designed for more active play. Children have much more space to move about and may have gross motor toys, such as tricycles or scooters. This environment gives opportunities for them to engage in active play and prompts them to do so. Furthermore, the expectations for children’s behavior are different across outdoor and indoor play. Although teachers would typically allow, even encourage, children to run, hop, or jump while outside, these behaviors would be discouraged while inside the classroom. Thus, the differences in how indoor and outdoor spaces are arranged, as well as the different expectations for behavior across them, mean that children will naturally engage in different amounts of physical activity in them. As a result, young children are more active outdoors than they are indoors [9,14].

### 1.2. The Current Study

Given the recommendations from USDHHS [6] and the IOM [1] that children average 15 min of physical activity per hour and the reality that many centers provide only limited outdoor time, the aim of this study was to understand how long it takes children to reach recommended levels of physical activity while at preschool. The key questions of this study were: How many children achieve the recommended 15 min of activity in the first 15 min of outside play? How long does it take children, on average, to reach 15 min of activity, and does this vary by whether they are indoors or outdoors? How much of children’s activity occurs in sporadic bursts? Do children engage in a spike of activity when they first get outside and then level off for the remainder of the time? The answers to these questions can help determine the length of play time needed, either indoors or outdoors, for children to achieve 15 min of activity per hour. This study presents visualizations to illustrate to researchers and practitioners the patterns of children’s physical activity and how they differ between indoor and outdoor settings.

## 2. Materials and Methods

### 2.1. Procedure

This study took place at a university-based laboratory school in the southwestern United States that offers a half-day preschool program and a full-day kindergarten program. Children were eligible for the study if they attended the preschool or kindergarten program at the study site during the fall of 2019. Children were included in the study if their parents gave written consent for their participation and if the children themselves gave verbal assent.

As in many early childhood settings, indoor activities in this setting consisted primarily of different classroom centers including arts and crafts, puzzles, literacy center, block building, and dramatic play. In contrast, the outdoor playground had open spaces for children to run after one another, skip, jump, and engage in other gross motor activities. Children also engaged in make-believe play, explored the outdoor environment, and engaged with toys and with playground features such as slides and sandboxes. Both indoors and outdoors, children were free to choose the activities in which they engaged and to shift freely between them. Each child participating in the study wore an Actigraph GT3X (Actigraph, Pensacola, FL, USA) accelerometer, a small, lightweight device widely used in studies of human activity and movement [18]. Accelerometers detect movement in three-dimensional space by measuring changes in acceleration values. They are thus able to detect movement across a range of physical activities (e.g., sitting, running, jumping). The device is worn with an elastic belt, allowing participants to engage in regular, unimpeded movement. The device does not have any display that provides feedback to participants or data collectors in the field.

The devices were attached comfortably around the children’s waists and over their clothes by their parents or by their classroom teachers at the beginning of the school day. Prior to collecting the data for the present study, we collected pilot data with the children for a week, which allowed them to become used to the devices and minimize any novelty effects that might influence their activity levels. These pilot data were excluded from analysis. For the current study, we selected accelerometer data from roughly an hour of indoor play and an hour of outdoor play collected on two separate occasions; a research assistant was present and recorded whether the children were playing indoors or outdoors. Though we aimed to collect an hour of data both outdoors and indoors, children wore the devices for about 52 min, on average, during each period on separate days. The devices were set to record data in 1 s epochs. This procedure was approved by the institutional review board (protocol number 2019-02-0042) at the researchers’ university.

### 2.2. Measure of Activity Level

We employed a conversion equation developed by Pate and colleagues [19] to convert raw activity counts into intensities of physical activity for preschool children. To do so, we first grouped the data into intervals of 15 s. The conversion allowed researchers to align activity counts from Actigraph devices to activities of known physical intensity (e.g., sitting, walking, running). For children of preschool age, Pate and colleagues [19] determined that activity counts greater than 200 in a 15 s period indicate non-sedentary activity (i.e., light, moderate, or vigorous physical activity). In this naturalistic study, children arrived at school at slightly different times. This meant that they did not all wear the devices for the same amount of time. To account for these differences, we used wear-time validation procedures to identify time periods during which children were very likely to not be wearing the devices (i.e., an extended period of 0 values at the start of the period observed [20]). We also excluded data if the child wore the device for less than 20 min or if the data were otherwise unusable (e.g., due to device malfunction).

### 2.3. Analytic Approach

To calculate children’s cumulative amount of time spent in light, moderate, or vigorous activity, we flagged each second in the observation periods that was preceded by a 15 s period with a total accelerometer count greater than 200, indicating that it represented physical activity (light, moderate, or vigorous) [19]. These calculations were performed in Stata 16 [21]. This process retained the cutoff developed by Pate and colleagues for 15 s intervals but allowed us to generate second-to-second cumulative activity totals. Although we calculated these in seconds, we presented results in minutes for ease of interpretation.

To address our research questions, we conducted several descriptive calculations. We first calculated children’s cumulative number of physically active minutes at five-minute intervals (i.e., after five minutes, after ten minutes) and then calculated the percentages of children who had reached 15 cumulative minutes of physical activity during both indoor activities and outdoor play at each of these intervals. To test statistical significance for the differences between activity in outdoor versus indoor play, we used multilevel regression models [22] with activity totals nested within the children. This allowed us to account for the dependencies among observations and use all available data. Finally, to understand how children’s activity is characterized by starts and stops, we plotted children’s cumulative activity totals over time, calculated the average rate at which children switched from sedentary to non-sedentary activities (and vice-versa), and identified the proportion of physically active time that occurred in bouts of less than 10 s, 20 s, and 30 s.

## 3. Results

A total of 67 children participated in the current study, contributing 315,061 s of activity data. Children were on average 4.51 years of age (SD = 0.75, range 3.15 to 6.12). Slightly less than half of the children were female (46%) and 73% were non-Hispanic White; 14% were Hispanic White; and 13% were either Black, Asian, or of another race. Among our sample, 34% attended the morning half-day program; 46% attended the afternoon half-day program, and 20% attended the full-day kindergarten program. Of the 67 children who participated in the study, 51 assented and had valid data for indoor activities, 50 assented and had valid data during outdoor play, and 35 children assented and had valid data for both. We report findings based on all available data but conducted a sensitivity analysis using only those children who had valid data both indoors and outdoors. The results of this sensitivity analysis were similar to those obtained from the full sample and are available upon request from the first author.

**Figure 1:**
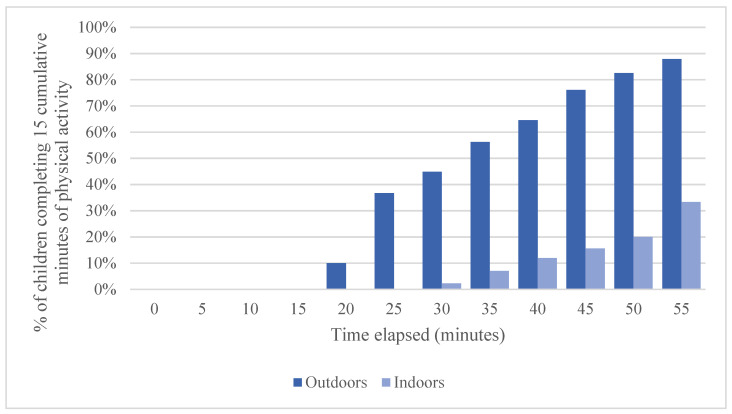
Percentage of children completing 15 min of physical activity during outdoor free-play versus indoor activities by elapsed time.

None of the children in our sample engaged in 15 cumulative minutes of physical activity during the first 15 min in which they were outside (0%; see Figure 1). Roughly half of the children in our sample (45%) reached this benchmark after 30 min. The percentage of children reaching this benchmark grew over the course of an hour, reaching 76% after 45 min and about 90% after 60 min of outdoor time. Regarding children’s physical activity indoors, 2% of children engaged in 15 min of physical activity after 30 min of center-based play indoors, 16% after 45 min, and 33% after 60 min (see Figure 1).

To understand which children met the 15 min-benchmark after 30 min of outdoor play, we examined the amount of activity by age or sex or by morning vs. afternoon attendance. The children who reached 15 min of physical activity during the first 30 min of outside time were about 6 months older, on average, than the children who did not, *t* (48) = 2.34, *p* = 0.02. We did not observe differences by sex ($\chi^{2}$ (1) = 1.67, *p* = 0.20) or by preschool attendance in the morning or afternoon ($\chi^{2}$ (1) = 0.08, *p* = 0.77).

**Figure 2:**
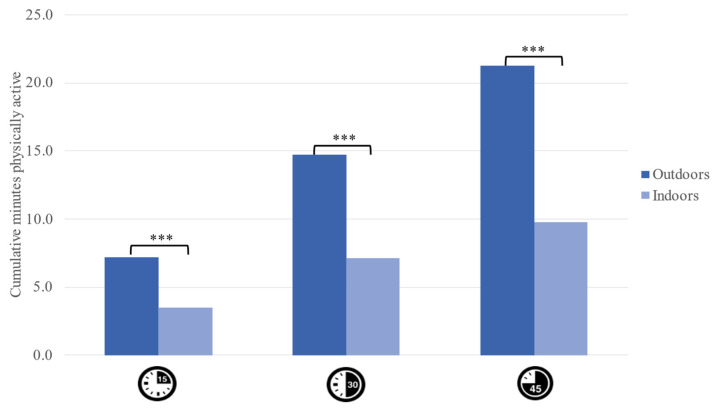
Mean cumulative minutes of physical activity in outdoor play versus indoor by 15 Min intervals. Note: *** *p* < 0.001.

The multilevel models revealed that, on average, children engaged in physical activity slightly less than half of the time that they were outside: 7.19 min after 15 min outdoors, 14.72 min after 30 min outdoors, and 21.23 min after 45 min outdoors. Children were, as predicted, less active indoors, engaging in 3.49 min of physical activity after 15 min of indoor activities, 7.11 min after 30 min indoors, and 9.78 min after 45 min indoors. Regression coefficients indicated that children consistently engaged in less physical activity indoors than outdoors (after 15 min; *b* = −3.70, 95% CI (−2.89, −4.62), *p* < 0.001; after 30 min: *b* = −7.62, 95% CI (−5.86, −9.37), *p* < 0.001; after 45 min: *b* = −11.45, 95% CI (−8.79, −14.11), *p* < 0.001; see Figure 2). In total, children engaged in physical activity half as often indoors (22%) as they did outdoors (45%; *b* = −23.48, 95% CI (−18.29, −28.67), *p* < 0.001). Children did not appear to engage in a spike of physical activity when beginning play outdoors. Instead, children’s average engagement in physical activity was fairly constant while outdoors, increasing by roughly 7 min during each 15-min interval (see Figure 2).

**Figure 3:**
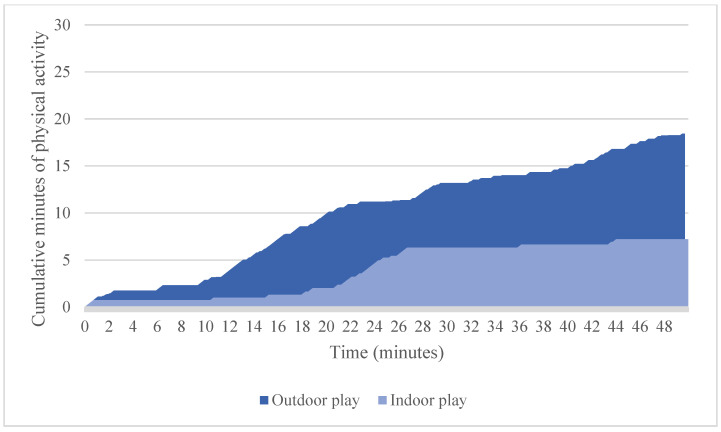
Cumulative minutes of physical activity during indoor activities for a 4-year-old male participant.

**Figure 4:**
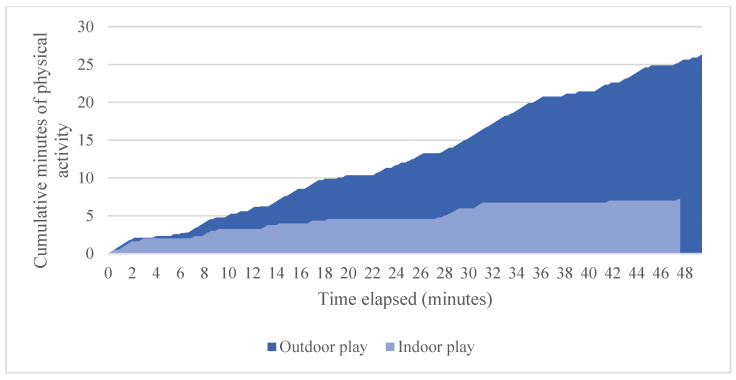
Cumulative minutes of physical activity during outdoor play versus indoor activities for a 5-year-old female participant.

To illustrate how children accumulated activity over the course of an hour, we plotted the cumulative total of indoor and outdoor activity for two participants (Figure 3 and Figure 4). Children showed starts and stops in their physical activities, as evidenced by plateaus in the figures. These starts and stops were relatively frequent, with children switching from sedentary to active play (or vice-versa) nearly twice per minute while playing outdoors (1.7 times per minute, on average) and about once per minute while indoors (1.09 times per minute, on average). Much of the children’s physical activity occurred in relatively short bouts while outside; that is, 29% occurred in bouts that lasted fewer than 10 s; 52% lasted fewer than 20 s; and 65% lasted fewer than 30 s.

## 4. Discussion

This study demonstrated that preschool children are not continuously active during outdoor play. Notably, no child in our sample reached the recommended 15 min of physical activity after 15 min of outdoor play. In fact, most children needed more than 30 min to reach this benchmark. The reason that 15 min of outdoor play did not equal 15 min of physical activity was that the children had frequent starts and stops and much of their activity occurred in relatively short bouts, as is evident in the examples graphed in Figure 3 and Figure 4. Children may pause during play to negotiate rules and roles with other children, make requests for sharing toys and resolve conflicts, all of which are critical for the development of social competence [23].

The preschool years are an important time to learn and refine fundamental movements and locomotor coordination. The health benefits of physical activity include increases in cardiorespiratory fitness, muscle strength, and bone density as health protective factors [2,3,6]. Even for young children, physical activity promotes a healthy weight status and bone health [4]. Physical activity is consistently linked with improved cognitive development as well as psychosocial and cardiometabolic health [5]. Although a preschooler’s physical activity depends on many factors such as age, ability, and physical maturity, it was not a surprise that the activity patterns of children in this study were both spontaneous and intermittent [24].

Even though adult-led activities can be effective in boosting children’s activity [25], educators should also consider how structured activities might constrain opportunities for peer interactions, conflict resolution, and child-directed play. From a practical perspective, many early childhood educators may not have training in structuring appropriate physical activity, meaning that adult-led programs might not be as effective as they could be, and student-directed activities may also be effective in promoting activity. For example, Montessori programs, which give a central role to child-initiated activities, promote more physical activity than do traditional preschool settings [15]. One way that adults can encourage children to engage in physical activity on playgrounds is to model activities, invite children to play, and resolve conflicts if necessary [26].

Consistent with other studies [9,11], our results suggest that young children are more active outdoors than indoors while at preschool. Providing sufficient outdoor time is therefore a critical component for encouraging children to engage in physical activity. Schools and other out-of-home settings may be especially important contexts for promoting activity because many young children do not get sufficient outdoor time at home: according to a nationally representative study, about half of preschool children are not taken daily by their parents to play outside [27].

Our findings indicate that periods of outdoor play are necessary to ensure that most children engage in the recommended amount of activity to remain healthy. These findings align with evidence from Head Start programs in the United States that children attending centers with at least 60 min of outdoor play each day showed a lower likelihood of obesity [13]. Future research might determine the ideal amounts and division of outdoor play time. For example, two 30 min breaks might prompt children to be more active than would a single outdoor play period of 60 min. Such an approach shows promise: A multi-site randomized trial in Australia demonstrated that having three outdoor play periods during the school day increased their physical activity in childcare relative to one continuous period [28]. Research in U.S. elementary schools suggests other potential benefits from dividing outdoor play time in similar ways. For example, recess at the beginning of the school day promotes attention, whereas recess just before lunch promotes fruit and vegetable consumption and reduces plate waste [29]. The optimal division and timing of outdoor time is an empirical question that future research could address.

### Strengths and Limitations

This study objectively assessed children’s physical activity using accelerometers, which minimized reporting bias. Children were observed naturalistically in their regular school environment and thus our findings are applicable to real-world preschool settings. Future studies could incorporate more schools and observe children for longer periods of time. The data for the current study were drawn from a larger project in which children’s physical activity was assessed along with other characteristics of their development. To reduce the study burden on participants, the children only participated on two occasions. That said, our finding that children were non-sedentary 45% of their time outdoors is consistent with a systematic review of accelerometer studies finding that children are non-sedentary 44% of the time during outdoor play [30]. This consistency suggests that the level of activity observed in our sample is typical of children of this age, giving us greater confidence in the generalizability of these findings. The fact that they emerged from a naturalistic rather than a controlled setting meant that they may also be generalized over other settings where children play and are active.

## 5. Conclusions

Our study illustrated that indoor free-play does not, on its own, provide sufficient opportunity for preschoolers to engage in physical activity consistent with health guidelines. During outdoor free-play, children engaged in physical activity sporadically and in bouts that lasted, on average, fewer than 20 s. Most children only reached the recommended level of physical activity after 30 min of outdoor play, and some children needed longer. Because of the intermittent nature of movement at this developmental stage, it is important that caretakers and educators understand how to organize children’s time to help them engage in the recommended amount of physical activity to stay healthy. Our findings suggest that preschool programs need to include at least 30 min of outdoor play to help young children meet health guidelines. Researchers should identify what activities, spaces, and school schedules best promote such physical activity.

## Data Availability

The data that support the findings of this study are not publicly available due to privacy or ethical restrictions. The data can be requested from the corresponding author.
